# Four years of free orthodontic care for vulnerable children: a community case study from the Caritas Dental Clinic in Rome

**DOI:** 10.3389/fdmed.2026.1863855

**Published:** 2026-08-13

**Authors:** Cristina Grippaudo, Ilaria Tucci, Elena Gimondo, Elisa Russo, Iole Vozza

**Affiliations:** 1 Dipartimento Universitario Testa, Collo ed Organi di Senso, Università Cattolica del Sacro Cuore, Rome, Italy; 2 UOC Clinica Odontoiatrica, Dipartimento di Neuroscienze, Organi di Senso e Torace, Fondazione Policlinico universitario A. Gemelli IRCCS, Rome, Italy; 3 Private Practice, Rome, Italy; 4 Dipartimento Scienze Odontostomatologiche e Maxillofacciali, Sapienza Università di Roma, Rome, Italy

**Keywords:** access to care, community dentistry, malocclusion, oral health inequalities, oral health-related quality of life, orthodontic treatment, social determinants of health, vulnerable children

## Abstract

**Background:**

Access to orthodontic treatment remains limited for many socially vulnerable children and adolescents due to financial, administrative, and social barriers. Despite the high prevalence of malocclusion and its potential impact on oral health and quality of life, few reports have described practical models aimed at improving access to orthodontic care for underserved populations. This study describes a community-based model developed at the Caritas Dental Clinic in Rome to provide free orthodontic treatment to vulnerable minors.

**Methods:**

This Community Case Study reports the implementation of a free orthodontic care pathway between January 2022 and December 2025. Patients were referred through Caritas social services, family homes, and therapeutic communities. Orthodontic treatment was provided free of charge through collaboration between volunteer orthodontists, postgraduate students, Caritas staff, and partner dental laboratories. Descriptive statistics were used to summarize patient characteristics and treatment activity.

**Results:**

A total of 133 patients were assessed for eligibility, and 93 initiated orthodontic treatment. The mean age of treated patients was 12.5 years; 55.9% were male and 44.1% female. Most patients were foreign-born (74.2%), while 25.8% were Italian. Forty patients (30.1%) did not proceed to treatment, mainly because of missed appointments and difficulties in maintaining regular follow-up. Fixed appliances represented the most frequently used treatment modality, often combined with interceptive and orthopedic approaches.

**Conclusions:**

The experience of the Caritas Dental Clinic demonstrates the feasibility of a community-based model for delivering free orthodontic care to socially vulnerable children and adolescents. Collaboration among healthcare professionals, volunteers, educational institutions, and social services may help reduce barriers to orthodontic treatment and improve access to care for underserved populations. Further studies are needed to evaluate long-term clinical outcomes and the broader impact of similar programs.

## Introduction

### Global burden of malocclusion

Malocclusion is a general term used to describe alterations in dental occlusion, sometimes associated with disharmonies in jaw growth. These clinical conditions have a multifactorial origin and are highly prevalent worldwide. Among oral diseases, malocclusion ranks third after dental caries and periodontal disease.

According to a 2021 study by Cenzato et al. (1), which analyzed data from different geographical areas, the prevalence of malocclusion varies considerably depending on the presence of clinical signs, ethnicity, and patient age, with reported values ranging from 39% to 93%. A 2022 systematic review by Lombardo et al. (2) examining the epidemiological distribution of malocclusions across continents reported the highest prevalence in Africa at 81% (95% CI: 64–98) and Europe at 71% (95% CI: 0.62–0.82), followed by the Americas at 53% (95% CI: 47–59) and Asia at 48% (95% CI: 34–62). No data were available for Oceania.

Environmental factors frequently involved in the development of malocclusions include respiratory disorders leading to predominant mouth breathing and the presence of deleterious oral habits. Numerous studies have investigated these factors, although their interpretation in clinical practice remains challenging due to the variability and severity of associated clinical signs. Katib et al. (3) provided a comprehensive analysis of oral dysfunctions and their negative impact on occlusal development. A recent randomized clinical trial evaluating the effects of nutritive sucking, including pacifier use, demonstrated that negative effects may occur even before the age of two years (4).

Another widely discussed issue concerns the possible relationship between dental caries and malocclusion. Demonstrating a direct causal relationship between these two conditions is challenging. Nevertheless, certain clinical situations suggest a possible association (5). Feldens et al. (6) reported a higher prevalence of caries in patients with evident malocclusion, suggesting that functional alterations and the increased difficulty in maintaining adequate oral hygiene may facilitate bacterial plaque accumulation. For this reason, the authors suggested that orthodontic treatment may also contribute to reducing the risk of caries. From the opposite perspective, Kolawole et al. (7) investigated the presence of malocclusion in Nigerian children with caries and poor oral hygiene. Their findings showed that dental crowding and crossbite were associated with caries, whereas open bite and increased overjet were correlated with gingivitis.

Malnutrition has also been identified as a potential risk factor for malocclusion in disadvantaged populations, as reported in India by Anand et al. (8).

Education and awareness regarding the prevention of oral diseases vary across ethnic and socioeconomic groups. Children from socially disadvantaged backgrounds show a higher prevalence of dental problems (9). Socioeconomic status also influences the likelihood of seeking dental care, including orthodontic treatment (10).

Proper oral hygiene is crucial for promoting and maintaining oral health, even among elderly nursing home residents. The study by Binello et al. (11) states that this is a largely ignored yet present problem, with between 50% and 75% of elderly nursing home residents in Lombardy included in the study experiencing oral health problems, mostly related to dentures, difficulty with oral hygiene, and tooth loss.

### Impact of malocclusion on quality of life

In addition to the functional consequences affecting dental occlusion and oral functions, malocclusion—particularly when it affects facial appearance—may expose patients to the risk of bullying and negatively affect self-esteem. Numerous studies have investigated this issue, and recent evidence suggests an association between malocclusion and reduced quality of life. However, this relationship remains debated, as self-perception is also influenced by factors such as age and gender. Disadvantaged living conditions may further contribute to the neglect of dental care, potentially exacerbating or prolonging the social impact of poor facial and smile aesthetics.

### Socioeconomic inequalities and access to orthodontic care

In Europe, access to orthodontic care for patients with socioeconomic vulnerabilities varies depending on the funding made available for dental care in different countries (12–19). Of the countries cited in articles on the state of dental care in Europe, only in Scandinavia is the treatment of malocclusions in children and adolescents covered by the national health system. In many countries, access to publicly funded orthodontic treatment is determined through the assessment of malocclusion severity using standardized orthodontic indices. These instruments are intended to prioritize treatment for patients with the greatest clinical need and to allocate healthcare resources more efficiently. Among the most widely used indices is the Index of Orthodontic Treatment Need (IOTN) (20), whereas in Germany treatment eligibility within the statutory health insurance system is primarily based on the Kieferorthopädische Indikationsgruppen (KIG). Although both indices aim to identify patients requiring orthodontic care, differences in classification criteria and treatment thresholds may influence estimates of treatment need and complicate international comparisons (21).

### Existing healthcare policies in Italy

In Italy, the National Health Service has introduced several measures to facilitate access to care for vulnerable individuals (18). With regard to orthodontic treatment, vulnerable minors may receive low-cost orthodontic care if their clinical conditions fall within categories 4 or 5 of the Index of Orthodontic Treatment Need (20), as established by the Prime Ministerial Decree of January 12, 2017. Patients must possess a national health card and are required to pay for orthodontic appliances. Costs vary according to regional regulations and the tariffs applied by public healthcare facilities providing the treatment.

### Vulnerable children and barriers to treatment

Two studies conducted in the United States (22) and in the United Kingdom (23) highlighted limitations in public dental care systems in providing orthodontic treatment to disadvantaged patients. A study published in England in 2019 highlights the difficulties of accessing orthodontic care for deprived patients, despite the social dentistry measures present in the area (24). Another article regarding the situation in France concludes by stating the presence of inequalities for orthodontic treatment, in relation to social status, annual income, supplementary insurance and residence area (25).

A 2020 study by Nakhleh et al. (26), which analyzed factors influencing the duration of orthodontic treatment—including socioeconomic variables—identified maternal support as the most important factor associated with treatment adherence. In this context, social difficulties may not interfere with orthodontic treatment to the same extent as in other behavioral domains among disadvantaged adolescents.

Also in Italy not all vulnerable minors are able to access orthodontic care. In Italy, and particularly in the city of Rome, some children do not yet possess a national health card because they are awaiting the completion of immigration procedures, while others lack the financial resources necessary to cover even the reduced cost of orthodontic devices. Furthermore, various forms of social hardship—such as foster care placement or residence in social care institutions due to severe family circumstances—may further complicate access to treatment. Public healthcare services often require administrative procedures that may be difficult to navigate for individuals living in conditions of social disadvantage or poverty.

As a consequence, many vulnerable children are unable to receive orthodontic treatment and may experience progressive worsening of their dental condition (27).

### Knowledge gap

The existing literature, to our knowledge, does not present solutions to address the problem of access to orthodontic care in countries that are unable to fully cover the demand from vulnerable patients.

### Study aim

The aim of this article is to present the outcomes of a replicable model for the reception and delivery of orthodontic care implemented since November 2020 at the Caritas Dental Clinic (COC), where treatment is provided completely free of charge for patients, including those who do not possess a national health card. The present study may also contribute to a more comprehensive understanding of disparities in access to orthodontic care by examining treatment demand in relation to socioeconomic disadvantage. Furthermore, the findings may help inform the development of targeted care pathways and support strategies aimed at overcoming barriers to access and facilitating the delivery of appropriate orthodontic treatment to underserved populations.

### Materials and methods

#### Study design and setting

This is a Community Case Study to describe a behavior adopted to overcome the barriers to access orthodontic treatment for vulnerable minors in Rome, Italy.

The model of reception and orthodontic care for vulnerable minors was initiated thanks to an agreement between the Caritas Dental Center of Rome and the Master's Program in Orthodontics at the Catholic University of the Sacred Heart in Rome. The Master's Program helped equip the facility with all the instruments and consumables needed for treatment, while removable appliances are provided free of charge by some dental laboratories. The costs of other dental care and the running of the clinic are covered by Caritas.

#### Participants

The Caritas Dental Center employs secretarial staff, volunteer doctors, and experienced orthodontists, coordinated by on-site clinical tutors. All volunteers and Caritas staff have been carefully selected and have received specific training on the project's objectives and the appropriate approach to working with people in socioeconomic difficulty. Volunteers offer their services free of charge.

Patient referrals are made by parents or caregivers of minors who have visited the Caritas Center, located in parishes, to request their children's inclusion in an orthodontic treatment program because they are unable to afford the cost of orthodontic treatment at any other paid center. Caritas Listening Centers are run by volunteers who have completed training in welcoming and listening to people in need. They serve immigrants facing financial hardship or language barriers, who often struggle to navigate Italian bureaucratic procedures. They also serve Italians experiencing poverty and seeking financial support.Some minors are referred to us from Family Homes, which house minors whose relatives have lost parental rights or are illegal immigrant orphans. Here too, patients may be Italian or foreign.

### Clinical assessment

Orthodontic care at the Caritas Dental Clinic was introduced in November 2020, following an agreement established for the clinical internship of the Master's program in Orthodontics and Gnathology at the Catholic University of the Sacred Heart. During 2021, procedures for recruiting and planning orthodontic patient care were developed and the necessary materials were purchased. In the study, we report clinical data from January 2022 to December 2025. We selected this time period because it follows the start and coordination of orthodontic activities, and also follows the period of the COVID pandemic, which negatively impacted medical activities in general.

The age of patients ranges from 6 to 18 years, with rare exceptions.

Patients come from a wide variety of ethnicities, as Rome attracts immigrants from all over the world. There is no discrimination based on gender, religion, or other factors.

Treated patients include children or adolescents with malocclusion of any shape and degree.

Having a mild malocclusion is not a reason for exclusion, a patient is also accepted based on the potential benefit orthodontic treatment can bring to their self-esteem and whether the problem alters aesthetics or poses a risk of worsening as the occlusion develops.

One reason for exclusion may be failure to ensure maximum adherence to treatment. This is a critical factor, as some patients live in challenging family or social conditions, may be under guardianship, or experience housing instability, all of which may affect treatment adherence. Given the prolonged duration of orthodontic therapy, patient compliance is essential.

Some patients delay starting treatment if they first need to treat cavities or improve their oral hygiene. In this case, they are referred first to pediatric dentists and dental hygienists.

#### Treatment procedures

At the first outpatient visit, most orthodontic patients present with evident malocclusion. A minority had previously started orthodontic treatment but discontinued care due to migration to Italy or financial constraints that prevented continuation within the public Italian healthcare system.

At baseline, patients are assessed according to a standardized protocol:

1. Collection of demographic and social information. This information is for internal Caritas use only.
2. Medical history assessment;
3. Clinical evaluation to determine the need for orthodontic treatment, using age-appropriate indices [Roma Index (28), Baby Roma Index (29) or IOTN (20)]. The indices of need for orthodontic treatment are assessed by two experienced orthodontists, who have been calibrated in previously published studies (28, 29). The indexes are used more for information purposes on the orthodontic problems treated in the practice, than for selecting patients.

During visits, language barriers with immigrant patients in Italy are overcome with the help of translations and language mediators when available.

All treatments are preceded by a planning phase, including analysis of diagnostic radiographs (panoramic and lateral cephalometric radiographs). Intraoral and extraoral photographs, as well as digital scans of the dental arches, are obtained before treatment initiation. Clinical cases are documented in PowerPoint presentations and discussed collectively by postgraduate students and clinical orthodontic tutors.

Diagnostic procedures are repeated at regular intervals during treatment or whenever clinically indicated to monitor progress and adjust the treatment plan.

Prior to treatment, parents or legal guardians receive detailed information about the proposed therapy and provide written informed consent.

When additional restorative treatments or extractions are required, their timing is coordinated with other dental specialties.

Clinical activities are supported by one administrative staff and one dental assistants employed by Caritas, alongside trained volunteers involved in reception and clinical assistance. The facility allocates two dental units for orthodontic care, available three mornings per month.

### Data collection

Patients are identified with a number to safeguard their privacy. Sensitive data is stored in the reception office, managed by Caritas staff.

Clinical photographs are stored in a database accessible with a password known only to the receptionist, and are backed up by Caritas’ information systems. Dentists keep a clinical diary in a patient file, stored in an archive in the reception office. The services performed are coded, so that statistical reports can be made.

### Statistical analysis

To provide a measure of the work carried out in the reported period, we present tables indicating the type of orthodontic activity and the volume of patients.

### Ethical considerations

Formal ethics committee approval was not required for this Community Case Study. Sensitive patient data is not considered. Furthermore, the clinic's policy requires that care be provided according to best practices.

## Results

**Figure 1:**
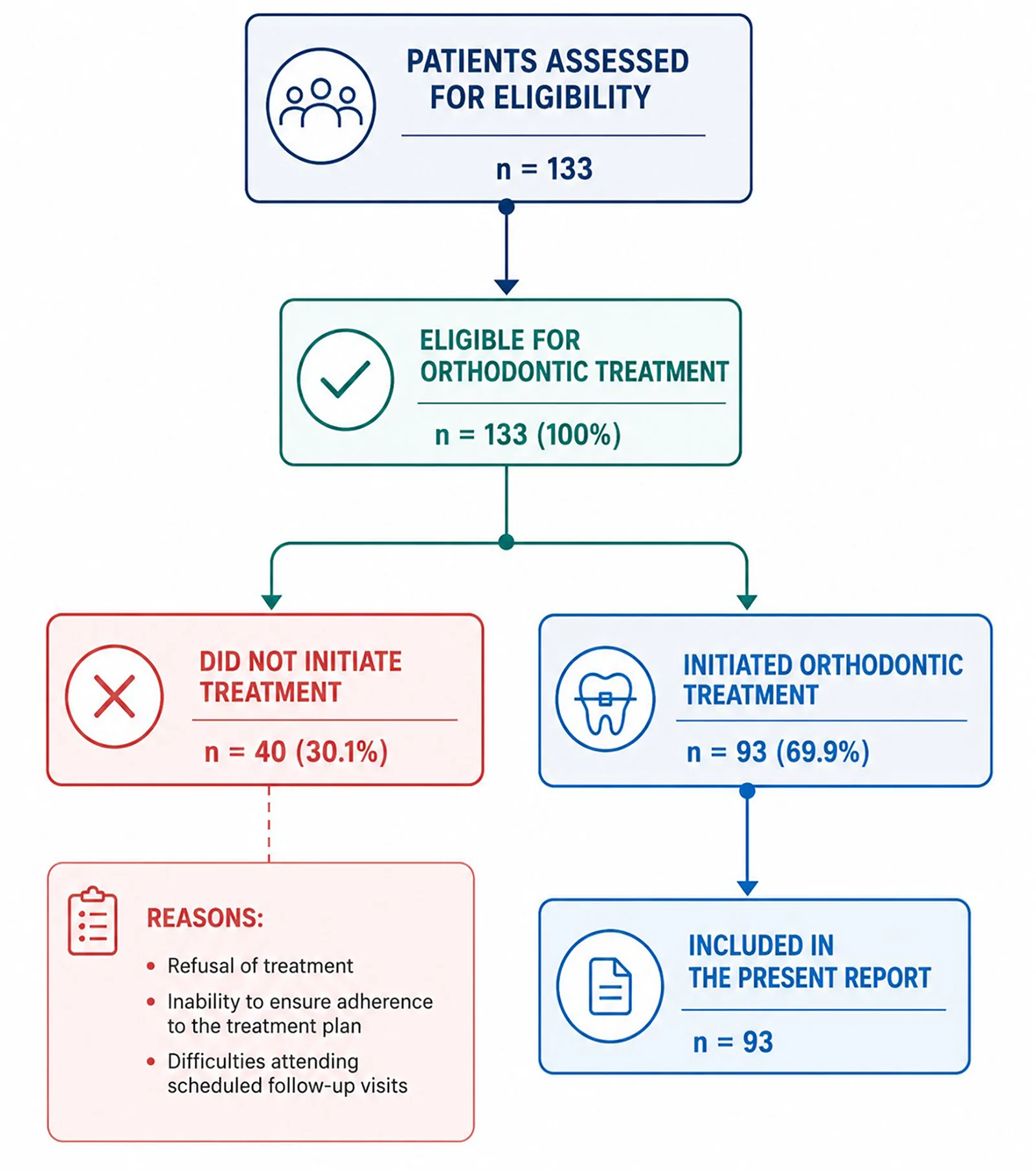
Patient flow-chart through the caritas orthodontic care pathway (January 2022–December 2025.

During the observation period (January 2022–December 2025), a total of 93 patients underwent orthodontic treatment at the clinic, with a mean of new 23 treated patients per year approximately. A total of 133 patients were assessed and considered eligible for orthodontic treatment. Of these, 93 patients (69.9%) initiated treatment and were included in the present report. Forty patients (30.1%) did not proceed to treatment because of refusal of treatment, inability to ensure adequate adherence to the treatment plan, or difficulties attending regular follow-up visits (Figure 1).

**Figure 2:**
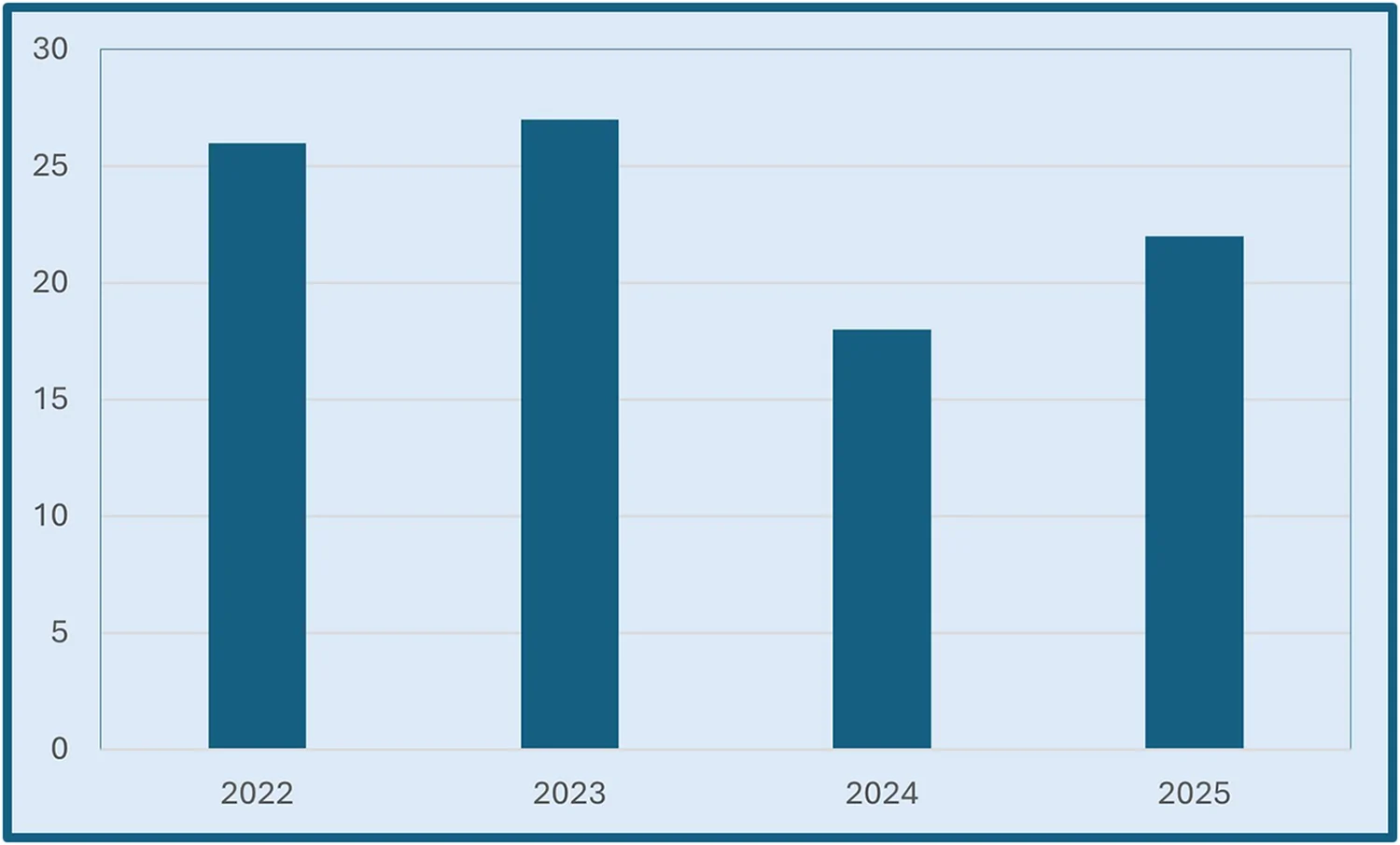
The graph shows the number of patients enrolled from 2022 to 2025. Until 2023, enrollment was constant and progressive, until the treatment offering was complete. Subsequently, the number of new patients depends on schedule availability.

During 2023, the necessary patient recruitment was completed based on the available space and time. Subsequently, newly enrolled patients filled the spaces made available by those completing treatment. Scheduling based on patient numbers is not possible, as the length of orthodontic visits varies greatly depending on the type of treatment. First-time visits decreased due to unavailability of scheduling space, which was already occupied by patients undergoing treatment (Figure 2).

Table 1 shows the baseline characteristics of the distribution by sex, age, and nationality.

**Table 1:** Baseline characteristics of patients receiving orthodontic treatment at the caritas dental clinic (January 2022–December 2025).

Variable	Value
Patients assessed for eligibility	133
Patients who initiated orthodontic treatment	93
Male	52 (55.9%)
Female	41 (44.1%)
Mean age	12.5 years
Italian nationality	24 (25.8%)
Foreign-born	69 (74.2%)

**Figure 3:**
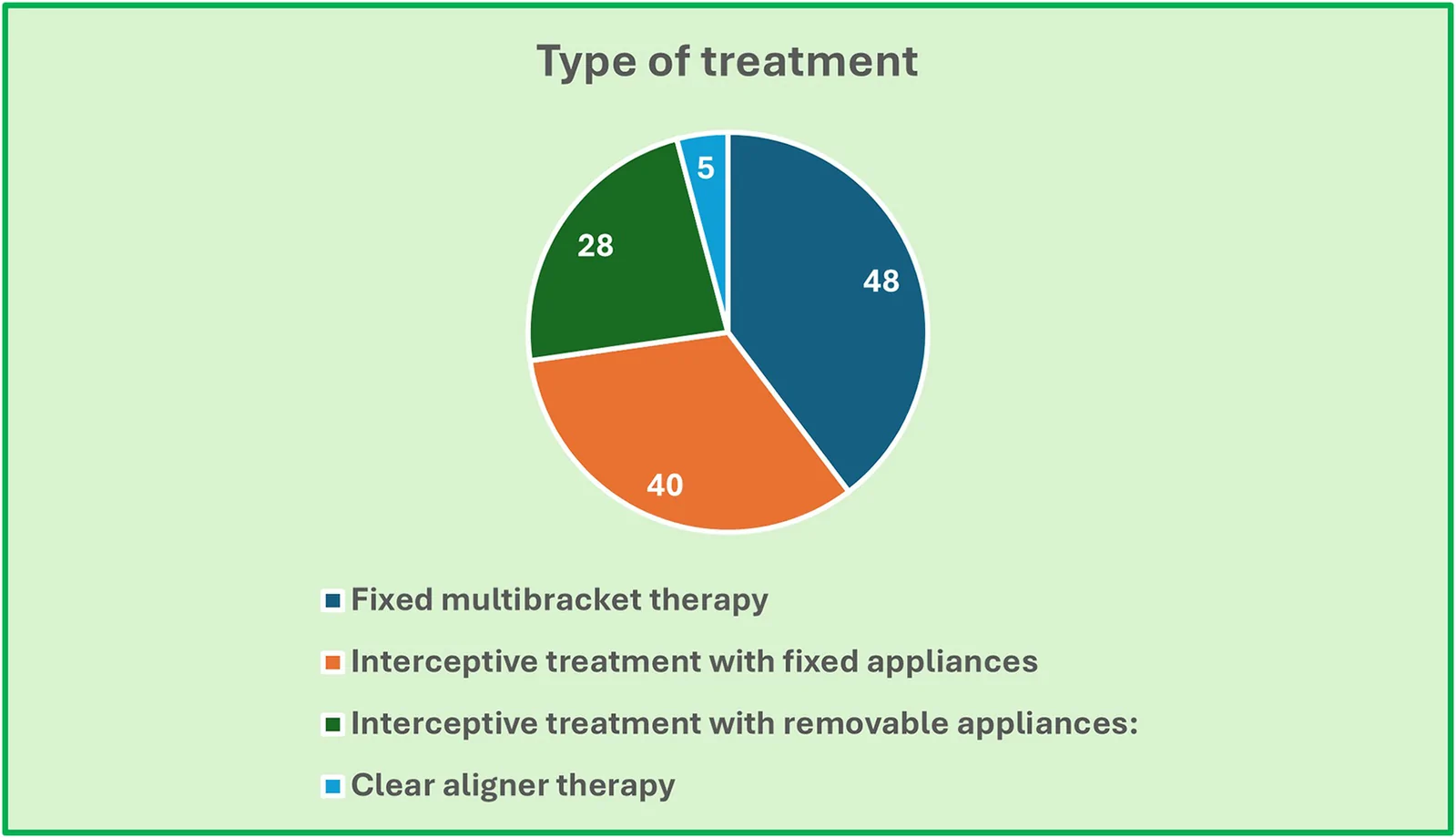
The graph shows the distribution of the types of orthodontic treatments provided.

A descriptive analysis of the treatments performed (Figure 3) showed that fixed orthodontic therapy represented the most commonly used approach, both as a standalone treatment and in combination with other appliances.

Interceptive and orthopedic approaches were frequently adopted; these included the use of rapid palatal expanders, McNamara-type expanders, quad helix appliances, Ni-Ti expanders, and removable expansion plates, often applied as a preliminary phase prior to fixed appliance therapy.

Functional and interceptive devices, such as active plates, lingual arches, space maintainers, and myofunctional appliances, were also utilized, mainly in early treatment phases. Only one case were treated with clear aligners.

## Discussion

Our experience providing orthodontic treatment to frail minors, in addition to the undeniable benefit of improving dental occlusion, has led us to make some social considerations.

During the implementation of the program, clinicians frequently observed that some families perceived orthodontic treatment as unaffordable or of lower priority compared with other social needs. Many parents have obvious malocclusions, and it is common for them to have lacked the education or opportunity to take care of their own oral health. Cenzato et al. (30) used a questionnaire to highlight the correlation between parents’ socioeconomic status and attitudes toward oral health maintenance in Italian patients.

The frail patients treated at the Caritas Dental Clinic belong to both Italian and foreign families. In the case of Italian patients, the parents very often have dental problems of their own, including malocclusion. It is common to observe people neglecting their oral health for economic reasons and due to the difficulties they face in everyday life. Some parents, in turn, experience a sense of vulnerability, and not all are able to support their children in their daily activities. In some cases, children are accompanied by guardians.

The situation is different for immigrant patients, where economic factors are compounded by the customs of their cultures of origin. Among some immigrant families, language barriers, differences in health literacy, and frequent mobility may occasionally complicate communication regarding treatment needs and continuity of care. These observations should be interpreted as practical challenges encountered during service delivery rather than as characteristics of any specific population group.

Parent supervision of minor patients undergoing orthodontic treatment has been identified as a factor that influences treatment outcomes and treatment times. Chawlowska et al. (31) conducted a questionnaire-based study on this topic, which highlighted the importance of parents’ education and social status, including in terms of adherence to clinical recommendations. Research by Chen et al. (32), conducted in Wuhan, China, and by AlMugairin et al. (33), conducted in Saudi Arabia, indicates that the maternal figure plays a key role in influencing children's progress and adherence to treatment. The research conducted in Saudi Arabia also highlights a gap between mothers’ level of knowledge of best practices for preventing dental diseases, including malocclusion, and their behavior. For example, knowing the benefits of early cessation of non-nutritive sucking does not lead to dissuading children from doing so.

For the immigrant patients we treat, we sometimes have to take breaks in treatment when they return with their families to their country of origin for varying lengths of time. In these cases the therapy time was extended.

Another recently highlighted psychological aspect (34) that can improve adherence to orthodontic treatment is a peaceful family environment. Unfortunately, this factor is lacking in some of the children we treat. Orthodontics for minors require collaboration, which depends on self-motivation, peer and authority influence, quality of life impairment and adaptability, perceived treatment progress, and pragmatic and recall issues (35).

The feasibility of the program is supported by the ability to provide orthodontic care to 93 vulnerable children over four years through collaboration among Caritas volunteers, orthodontic specialists, postgraduate students, and partner dental laboratories. Although formal outcome measures were not collected, the continuity of service suggests that this model may represent a sustainable approach for improving access to orthodontic care in underserved populations.

### Limitations

This Community case study is limited by its single-center design, relatively small sample size and descriptive nature. In addition, treatment outcomes were not formally evaluated, and the referral process may have introduced potential selection bias.

Information regarding treatment completion, loss to follow-up, and standardized distributions of orthodontic treatment need indices was not systematically collected and therefore could not be included in the present analysis.

## Conclusion

In our experience, we have observed that providing good orthodontic treatment to vulnerable patients requires more than simply offering free treatment; it requires motivational efforts equal, if not superior, to those undertaken with paying patients.

It is essential to establish a good relationship with the minor receiving treatment and with those accompanying them, in order to convey the feeling of welcome and support that underpins all medical and non-medical activities carried out in the clinic.

We conclude this report on the experience of orthodontic treatment for vulnerable minors by reiterating the importance of orthodontic treatment, not only for improving dental occlusion, but also for enhancing self-esteem, personal care education, and consequently preventing future dental diseases. Unfortunately, our experience is limited due to limited financial resources, but it is hoped that it can be replicated where possible.

## Data Availability

The original contributions presented in the study are included in the article/Supplementary Material, further inquiries can be directed to the corresponding author.

## Author disclaimer

The research does not require the approval of an ethics committee because patients are not the subjects of the experiment. In this article, we present the care organizational model we adopted to provide free orthodontic treatment to vulnerable minors who have difficulty accessing the same care in Italian public facilities. The numerical data we report, which correspond to the treatments provided, cannot in any way be linked to sensitive patient data. Patients are treated by expert professionals with uncompromising care, a prerequisite for acceptance as collaborators at the Caritas Dental Clinic.
